# Age affects pigeons’ (*Columba livia*) memory capacity but not representation of serial order during a locomotor sequential-learning task

**DOI:** 10.1038/s41598-021-96360-1

**Published:** 2021-08-25

**Authors:** Christina Meier, Parisa Sepehri, Debbie M. Kelly

**Affiliations:** Department of Psychology, University of Manitoba, P438 Duff Roblin Bldg., 190 Dysart Rd, Winnipeg, MB R3T 2N2 Canada

**Keywords:** Psychology, Animal behaviour, Cognitive ageing, Learning and memory

## Abstract

Aging affects individuals of every species, with sometimes detrimental effects on memory and cognition. The simultaneous-chaining task, a sequential-learning task, requires subjects to select items in a predetermined sequence, putting demands on memory and cognitive processing capacity. It is thus a useful tool to investigate age-related differences in these domains. Pigeons of three age groups (young, adult and aged) completed a locomotor adaptation of the task, learning a list of four items. Training began by presenting only the first item; additional items were added, one at a time, once previous items were reliably selected in their correct order. Although memory capacity declined noticeably with age, not all aged pigeons showed impairments compared to younger pigeons, suggesting that inter-individual variability emerged with age. During a subsequent free-recall memory test in the absence of reinforcement, when all trained items were presented alongside novel distractor items, most pigeons did not reproduce the trained sequence. During a further forced-choice test, when pigeons were given a choice between only two of the trained items, all three age groups showed evidence of an understanding of the ordinal relationship between items by choosing the earlier item, indicating that complex cognitive processing, unlike memory capacity, remained unaffected by age.

## Introduction

As we age, so does our brain, and with it many cognitive functions decline. Age-related degeneration of cognitive and memory capacities is well-documented for humans, and is evident even for healthy individuals^1,2^. The impact of aging on nonhuman animal cognition is less well explored^3^, but this knowledge is important for understanding common effects of non-pathological aging on cognition. The aging process affects every living being regardless of species, but the way in which it manifests in cognitive changes or impairments can sometimes differ greatly among individuals. Establishing how, and to what degree, aging can affect vital cognitive capacities is a first step in assessing the range of normal and abnormal impairments.

The simultaneous-chaining task^4,5^ is cognitively demanding as it requires subjects to reproduce a list of items in a specific sequence, with the only feedback provided regarding the correctness of a choice being the continuation of the trial. This task puts demands on an individual’s memory capacity, in terms of both reference memory to learn the sequence, and working memory to update the last choice made in order to determine the next required response. An individual’s memory capacity can be measured through the successive chaining of subsequent sequence items^6^. Each time a subject learns to respond correctly to a sequence of *n* items, another item is added to the chain (*n*+*1*). Using this method, pigeons have successfully been trained to reproduce lists of four to five items^4,5^.

In addition to the task’s adoption for evaluating memory capacity, it has also been selected as an assessment tool for cognitive capacity, and as such has been used to study a diverse range of species, from humans and apes to pigeons and chickens^4^. As suggested by the task’s name, successful acquisition of the sequence can be achieved through simple associative chaining, with a response to the first item serving as a cue to respond to the second item, and so on. However, a more cognitively complex solution would be to form a mental representation of the item order, whereby each item in the sequence is acquired not only relative to the immediately preceding and following items, but also in terms of its unique ordinal position within the sequence as a whole (e.g., is the item the first, second, third, etc.). One way to assess a subject’s ability to form a representation of order is during subsequent pairwise-choice tests, for which only two items of the learned sequence are presented together, and the subject is allowed a single choice. If item order had been represented, subjects would be expected to recognise the ordinal relationship between the two presented items even in the absence of the complete sequence, and accordingly to choose the item that appeared earlier in the trained sequence. However, if learning occurred based on associative chaining alone, subjects would be expected to perform well only when the first item is present, but to be unable to distinguish between any later items, as the cue to respond to either—a successful response to the preceding item—would not have occurred.

During early studies by Terrace and colleagues, pigeons largely responded in line with the predictions for an associative-chaining account, in that for trials in which one of the presented items was the first item of the trained sequence, that item was chosen correctly, and for trials in which the first item was not part of the choice, a choice was made at chance^5^. However, Terrace also found one notable exception to this pattern: when a choice included the last sequence item, the other item was reliably chosen, and the last item was avoided. Terrace interpreted these results by assuming that pigeons had learned the special status of the two items to which a response was required first and last, but that they otherwise did not possess a mental representation of the ordinal relationship between two list items^5^. More recently, however, Scarf and Colombo argued that pigeons are indeed able to form a mental representation of the sequence order, if not in terms of exact ordinal position then at least in terms of appearing earlier or later in the sequence^4^. Proposing that their previous inability to maintain the trained order during two-item tests was due to a contextual change resulting from presenting only two items rather than the full sequence, Scarf and Colombo suggested that this change may have disrupted the pigeons’ ability to relate the test to the training and transfer the task requirements accordingly. Taking this critique into consideration, in our current study we presented subjects with a free-recall test, as well as the conventional pairwise-choice test. During the free-recall memory test, all items of the trained sequence were presented, thus preventing the sudden perceptual change that arguably impeded a transfer from training to test. In addition, previously unseen distractor items were shown alongside the familiar training items. Instead of just one choice, subjects were free to make several choices, in order to measure both item recognition and adherence to the trained order.

During this study, we adapted the conventional computer-based simultaneous-chaining task to a physical environment, such that pigeons were presented with an array of six feeders, distinguished by different colours, that had to be visited in a predetermined sequence. A physical task, as opposed to a computer-based task, capitalises on the pigeons’ natural foraging behaviour to allow for rapid training of food-searching behaviour, eliminating the need to administer extensive pretraining of touchscreen use, avoiding associated technical errors, and separating individual choice items with sufficient space to increase response cost or otherwise prevent involuntary wrong choices caused, for example, by abrupt body or head movements. Learning has been shown to be accelerated in physical settings as opposed to computer settings in comparable tasks (e.g., for stop-signal tasks, pigeons required an average of 34 trials to acquire the task contingencies in a computer setting^7^ versus two habituation trials in a physical setting^8^).

During training, pigeons learned to visit a sequence of up to four coloured feeders in order while ignoring uniformly white feeders. Following acquisition, a non-reinforced pairwise-choice test and an equally non-reinforced free-recall memory test were administered. During the choice test, two coloured feeders were presented along with four white feeders; the colours had either formed part of the trained sequence or not. Specifically, the choices were between: (1) two coloured feeders that were part of the trained sequence, (2) one previously trained and one novel coloured feeder, or (3) two novel coloured feeders. Although no “correct” or “false” response was ever indicated to the pigeons, the test served to assess whether pigeons would, for case (1) above, have a preference for coloured feeders that had been presented earlier in the sequence those that had been presented later, (2) have a preference for coloured feeders that had been presented in the trained sequence above those that had not previously been seen, and (3) might show potential systematic preferences when neither option had been presented before. During the memory test, six differently coloured feeders were presented (those presented during the training phase and additional feeders with previously untrained colours); the test assessed whether pigeons would visit feeders that belonged to the trained sequence and, if so, would adhere to the trained sequence in the presence of distractors.

In summary, the goals of our study were twofold: first, to investigate potential age-related decline in memory capacity. To achieve this, we assessed the length of a sequence that subjects of different age groups could acquire with a reasonable amount of training. It was expected that subjects belonging to the youngest age group would readily acquire the full sequence of four items. By contrast, subjects of the oldest age group were expected to require significantly more training sessions to learn the correct response each time that a new item was added to the sequence, and it was also considered possible that they might fail to perform correctly when having to reproduce the full sequence of four items. Additionally, compared to young pigeons, older pigeons might not show a clear preference for trained items over novel items during the pairwise-choice test presenting one familiar and one unfamiliar item. Second, we wanted to elucidate the underlying cognitive mechanisms governing task acquisition for pigeons. To do so, we investigated whether a subject’s age influenced its ability to create a mental representation of the trained order, i.e., to recognize whether an item appeared before or after another item in the sequence. It was expected that younger pigeons would preferentially choose items in the order they were trained, whereas older pigeons might not show such preference during the pairwise-choice test with familiar items.

## Methods

### Subjects

Twelve locally sourced racing pigeons (*Columba livia*) completed this experiment. They were grouped into three categories according to their age at test: *young* (1 year of age; *N* = 4, 2 females), *adult* (6–8 years of age; *N* = 4, 2 females) and *aged* (15–17 years of age; *N* = 4, 1 female). The pigeons were housed in individual metal (60 × 60 × 38 cm length × width × height) or plastic (75 × 70 × 50 cm) cages in a colony room at the Department of Psychology of the University of Manitoba. The room was kept year-round at a temperature of 21 °C and a 12-h light–dark cycle with lights on at 0700 h. The pigeons’ weights were monitored daily and maintained, through controlled feeding of a mixture of yellow and green peas, oat groats, red milo, yellow popping corn, and white millet, at 90% of their free-feeding weight and had *ad lib* access to water and grit inside their cage. The adult and aged pigeons had previous testing experience in an experimental arena (individual experience ranged from being experimentally naïve to participating in as many as three experiments), but all were naïve to the specific procedures of this experiment. Pigeons in the young group were all experimentally naïve.

### Ethical note

This study was approved by the University of Manitoba’s Local Animal Use Committee (protocol number F18-042) in accordance with the Canadian Council on Animal Care and the ARRIVE guidelines.

### Apparatus

**Figure 1:**
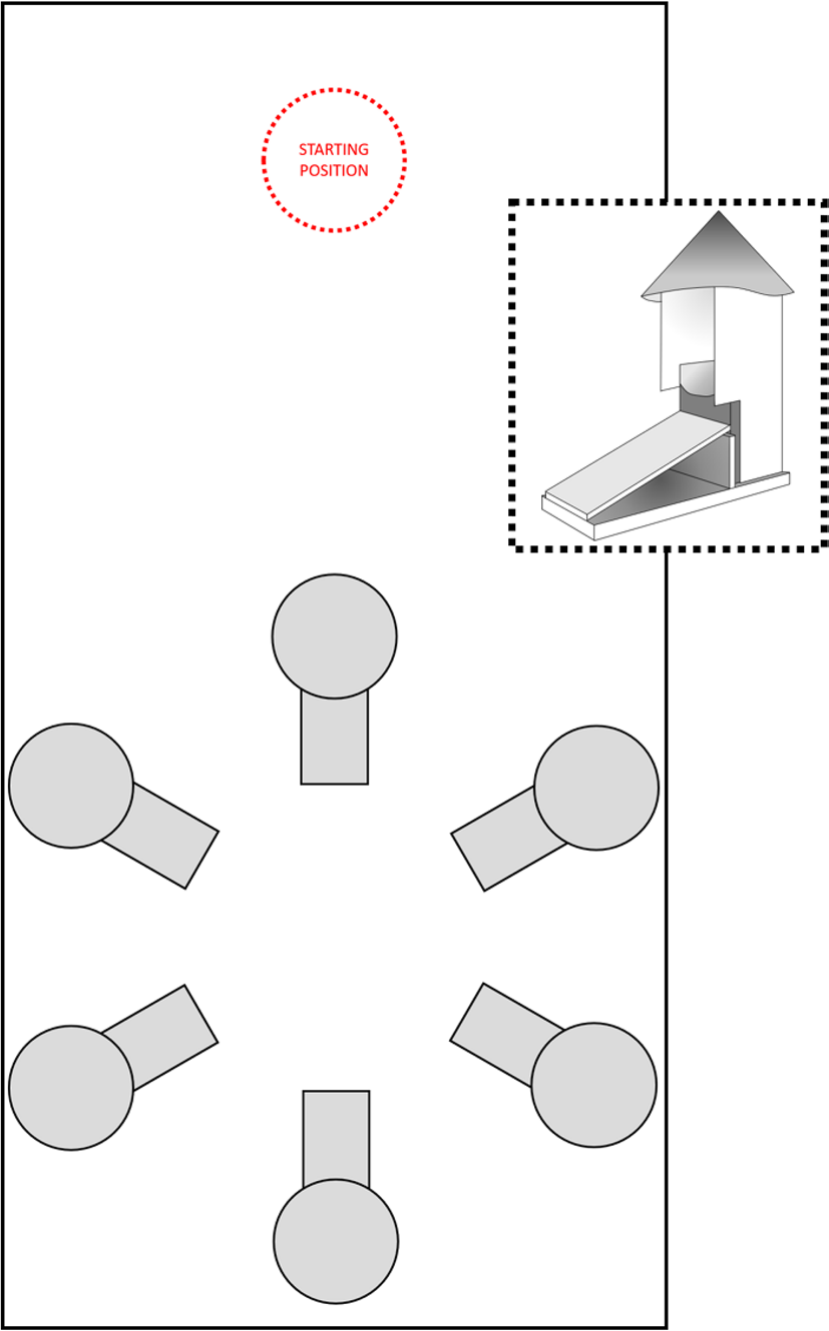
Top-down view of the inside of the experimental arena. The six feeders (in grey) were placed in a circular array in one half of the arena. The pigeon entered at the opposite side of the arena (the approximate location is marked in red). The insert in the broken-line box shows a 3D model of a feeder.

Training and testing sessions were conducted in an enclosed rectangular arena made of Styrofoam. The walls (60 cm tall) were lined with white Con-Tact paper to permit cleaning. The base (200 × 100 cm length × width) was covered with black rubber flooring. White plastic curtains enclosed the arena to block access to external visual cues. Six feeders were placed in a circular array inside the arena and fixed to the floor with Velcro (see Fig. 1). Each feeder consisted of a wooden base (10 × 10 × 10 cm length × width × height) and a wooden ramp covered with sandpaper (10 × 20 cm base with a 26.6° incline) leading up to the base. A plastic cup (6 cm diameter) was attached to the inside of the base. To make the inside of the feeder only visible from the top of the ramp, the three sides of the base facing away from the ramp were lined with white paper (30 cm height), and a white foam cone was attached to the top of the paper barrier. The white cones could be replaced by equally-sized cones of different colours to make each feeder visually distinct (herein these coloured cones are referred to as *features*). A centrally-mounted Logitech HD Webcam C270, connected to a Dell desktop computer (running 64-bit Windows 10 Enterprise) in the adjacent room, was suspended from the ceiling for recording of trials. Two Conair white noise generators were placed external to the arena, at opposing corners, to mask sounds.

### Procedure

#### General procedure

A pigeon was transported from the colony room to the procedure room in an opaque white transport container, which also served as a holding container between trials. At the beginning of each trial, the lights in the procedure room and arena were extinguished and the pigeon was removed from the holding container and placed into the arena at the starting position (see Fig. 1). The experimenter exited the procedure room and remotely illuminated the procedure room lights, which delimitated the start of a trial. Once the pigeon completed the choice requirement (see below) or ten minutes elapsed, whichever occurred first, the lights were extinguished, and the pigeon was removed from the darkened arena and returned to the holding container where it remained while the arena was prepared for the next trial.

#### Feeder training phase

To train the pigeons to retrieve maple peas from the feeders, a single feeder with a white cone (herein referred to as a *white feeder*) was located in one of the six possible array locations (with location counterbalanced across trials). Five maple peas were placed at each of the following locations: in the plastic cup inside the feeder, on the ramp, and on the floor of the arena at the base of the ramp. With training, the total number of maple peas was gradually reduced from fifteen to two, and only placed inside the feeder. Pigeons were given a maximum of 60 min to consume the maple peas. A trial ended 30 s after the last maple pea was consumed, after which the arena was re-set and a new trial started. As each daily session lasted a maximum of 60 min, a pigeon could receive between one and twelve trials per daily session. Pigeons experienced trials with a single feeder until all maple peas were consumed within 5 min of trial start, for a minimum of 12 trials in total. Next, pigeons were presented with all six identical feeders arranged in the circular array, with one maple pea inside each feeder. Pigeons progressed to the Memory Training phase once every feeder was visited within the first 5 min of a trial for 5 consecutive trials.

#### Memory training phase

A daily training session consisted of 10 trials, with the six feeders positioned in the circular array formation. Across four distinct stages, the pigeons were trained to visit four of the six feeders in a specific sequence, as indicated by distinctly coloured features (i.e., red, green, blue, yellow, orange and purple; herein referred to as a *sequence item*). The order in which the feeders had to be visited was consistent for each pigeon, but pseudo-counterbalanced among the pigeons such that each one of the six colours was assigned to be the first item in the sequence for two pigeons. The location of each sequence item within the array was randomised across trials so that only the colours on the feeders, but not the feeder locations within the arena or relative to each other, indicated the correct sequence.

Originally, the stages described below were administered with all six coloured features placed on the feeders. However, although all subjects quickly passed the first training stage, during which only the first item in the sequence was reinforced, acquisition of any following memory items appeared hindered by this procedure. In particular, following a successful visit to the first item, the subjects stopped exploring the arena and were reluctant to visit any additional feeders, as visiting them had previously not been reinforced. For the majority of subjects, the transition to training stage 2 resulted in reduced attention to the previously learned first item. Following ten sessions of continued decline in performance by all subjects, we revised the training procedures to encourage exploration of the area after the first item had been visited. Thus, the entire training phase was restarted from stage 1 for all subjects as follows.

**Figure 2:**
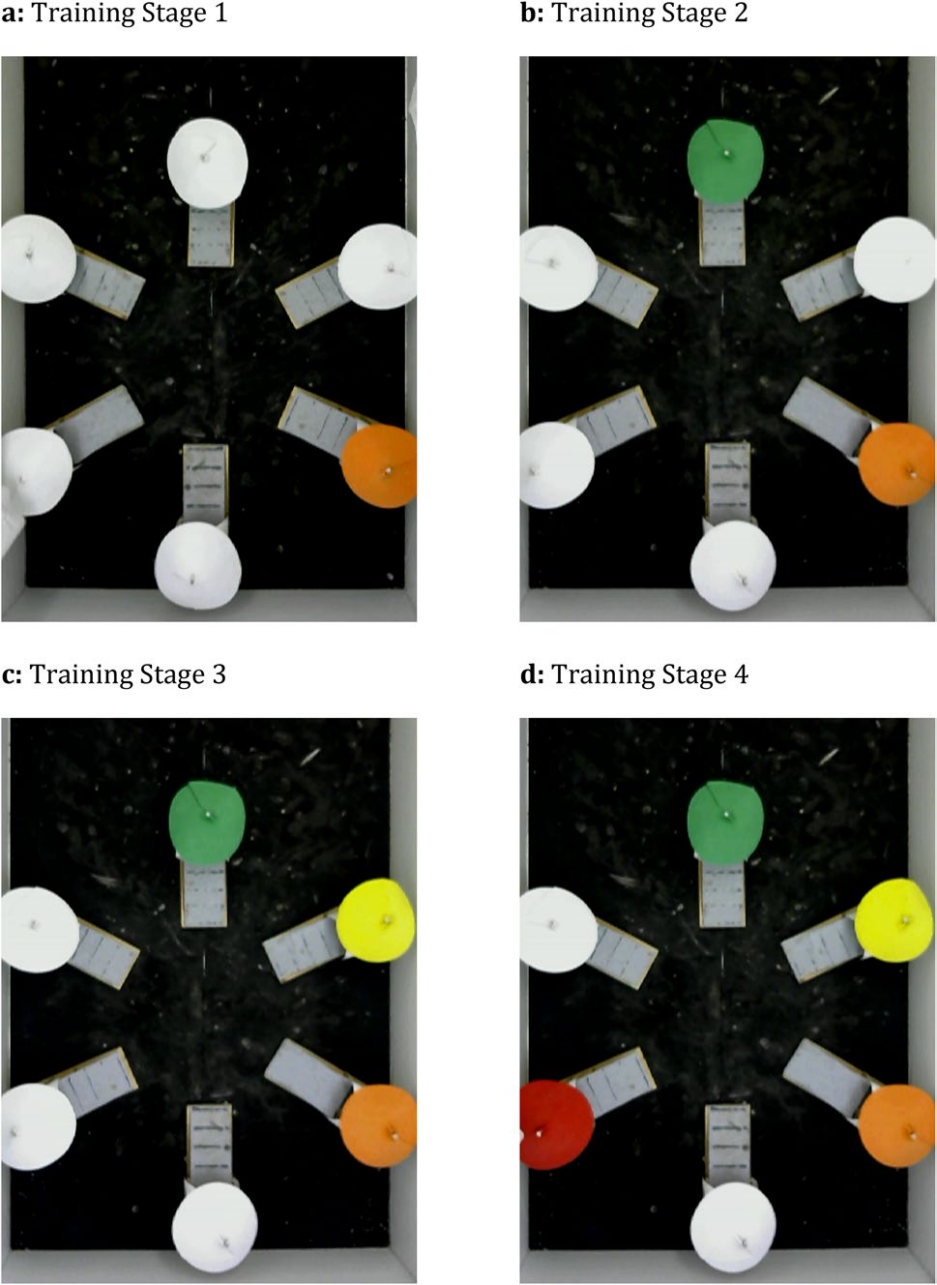
Examples of a trial in each memory training stage. The location of each sequence item (coloured feeder) changed between trials as to not systematically reinforce a certain location. The sequence shown here is for illustrative purposes.

During the adjusted memory training stage 1 (TS1; Fig. 2A), pigeons were presented with five white feeders and the first sequence item. Each successive training stage differed from the previous in that the next new sequence item replaced one of the white feeders in the array. Whereas the white feeders were always non-reinforced, during TS1, four maple peas were placed into the first sequence item; the pigeon was allowed to inspect any feeder. During training stage 2 (TS2; Fig. 2B), three maple peas each were placed into the first and second sequence items. If the pigeon approached any feeder before visiting the first sequence item, the lights were extinguished immediately to indicate an incorrect response and the trial ended. If the pigeon successfully visited the first sequence item, it could inspect any other feeder. During training stage 3 (TS3; Fig. 2C), two maple peas each were placed into the three sequence items. Only after the pigeon had approached the first and second sequence items, it was allowed to inspect any feeder. During training stage 4 (TS4; Fig. 2D), two maple peas each were placed into the four presented sequence items. Only after visiting the first, second and third items in sequence, the pigeon was allowed to freely inspect any feeder. During each stage, a trial ended with the extinction of the lights after the pigeon consumed all available peas and exited the final feeder, or if the pigeon visited the feeders in any other order than described, or after ten minutes of inactivity. Training progressed to the next stage once a pigeon consistently visited all presented sequence items in the correct order without visiting any other feeders in at least seven out of ten trials of a session, for two consecutive sessions. In Terrace’s study, pigeons acquired the first two items within 10 training sessions, the third item within 30 training sessions and the fourth item within 50 training sessions^9^. Based on these numbers, we estimated that each training stage could reasonably be expected to be acquired within 60 sessions. If a pigeon failed to advance from one training stage to the next within 60 sessions, it was considered to have reached the maximum number of sequence items it could memorise, and proceeded to the Testing phase.

#### Testing phase

Two tests were administered in alternating blocks of three sessions: a choice test, consisting of nine sessions, and a memory test, consisting of a minimum of six sessions. The tests served to examine the successful encoding of the trained sequence items, both regarding their identity (colour) and their position within the sequence. To this effect, the previously trained items were presented alongside novel items of the colours not used during the subject’s training.

*Choice test* This test recreated the pairwise forced-choice tests conducted in previous studies on sequence learning to determine whether there was evidence of a mental representation of the trained order. Each session consisted of five reinforced trials, which were identical to training trials during the final stage a pigeon had reached, and five non-reinforced test trials, presented in alternating fashion. During test trials, two feeders had coloured features. All 15 possible combinations of the coloured features (four colours presented as sequence items during training, plus two novel colours) were presented once per three-session block, resulting in a total of 45 test trials. Test trials ended after the first visit to any feeder.

*Memory test* Accounting for the critique by Scarf and Colombo^4^ regarding the disruptive effect of pairwise tests on performance potentially being due to the change in presentation of the sequence items, we additionally incorporated an open memory test presenting all sequence items and novel items together. Each session started with at least two reinforced trials, which were identical to training trials during the final stage a pigeon had reached. Following two correct baseline trials, a non-reinforced test trial was administered, during which all six (sequence and novel) items were shown in a predetermined, randomised order. Test trials ended after the pigeon made four visits, including repeat visits to the same feeder. Memory test sessions were continued until a pigeon had completed ten test trials. Due to the criterion of two correct baseline trials preceding any test trial, each session could contain between zero and three test trials.

### Data collection

Data collection was prematurely terminated due to restrictions put in place by the University of Manitoba in response to the Covid-19 pandemic. Two young subjects had completed 30 and 44 sessions of TS4, respectively, and one aged subject had completed 4 sessions of TS4 when the experiment was ceased; only their data from the completed TS3 were included in the analysis to provide an accurate representation of learning. One adult subject (Orange 11) was in the middle of the test phase when data collection was suspended; for this subject, all completed test sessions were included in the data analysis (providing data for 35 of the 45 Choice Test trials and 3 of 10 Memory Test trials).

### Data analysis

Given the format of the training data, we ran a linear mixed model (LMM) on the number of training sessions required to complete a training stage, with the simple factor Age Group (young, adult, aged) and the polynomial factor Training Stage (TS1, TS2, TS3, TS4), the hatch year of subjects as an unscaled covariate and pigeon identity as the cluster variable assuming correlated effects. Planned pairwise comparisons of performance were conducted as *t*-tests, the respective significance values reported are Bonferroni-corrected. Degrees of freedom were calculated using the Satterthwaite approximation. A LMM simple-effect analysis was included to investigate the presence of age-group differences within training phases. Lastly, a Kruskal–Wallis non-parametric one-way ANOVA was performed to assess potential age-group differences for the highest daily performance score achieved during the first half of training in TS4.

Analyses of test performance were carried out separately for the two groups of pigeons for which sequence item 3 or 4 had been the last trained item. For the Choice Test, choices between two previously trained items were assessed by conducting Friedman non-parametric repeated-measures ANOVAs on the percentage of trials during which a subject chose the item that had appeared earlier in the sequence, for each combination of trained sequence items. Choices between a trained and a novel item were assessed by conducting Friedman non-parametric repeated-measures ANOVAs on the percentage of trials during which a subject chose a previously trained item over a novel item. To meaningfully analyse performance in the Memory Test, we considered the correct execution of smaller “chunks” within the trained sequence, specifically, a subject’s tendency to choose sequence item 1 first before visiting any other item, to choose at least two sequence items in their correct order (i.e., 1 followed by 2, 2 followed by 3, or 3 followed by 4, regardless of which item was chosen before or after this isolated combination of two items), or to choose the final trained item last. Friedman non-parametric repeated-measures ANOVAs were performed on the percentage of trials during which a subject completed each of these chunks. For any of these ANOVAs, pairwise post-hoc Durbin-Conover comparisons between individual factor levels were performed if an analysis indicated a significant main effect. All analyses were carried out in jamovi version 1.2^10^.

## Results

### Memory training

**Figure 3:**
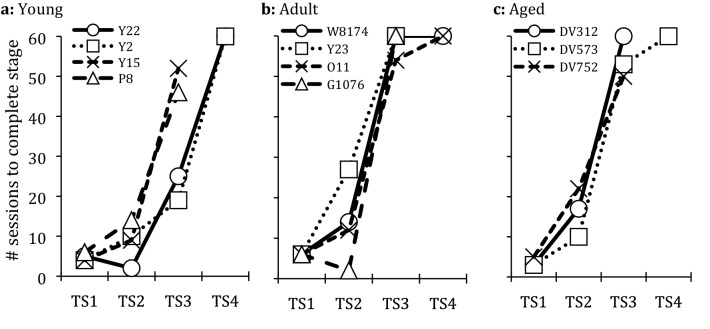
Number of sessions required to reach completion criterion in each training stage (TS) for each individual per age group. Denominations in the figure legends refer to individual pigeons.

Individual performances are displayed in Fig. 3; mean performance per age group is presented in Table 1. All 12 subjects successfully passed TS1 and TS2, with the exception of one aged subject that failed to continuously search the feeders during TS2 and was removed from the experiment. First performance differences emerged in TS3, as only two subjects each in the adult and aged group reached the training criterion, in contrast to all four subjects in the young group that passed this stage. Finally, none of the subjects that entered TS4 were able to pass the criterion within the number of training sessions they received, leading to an observable ceiling effect for TS4 in Fig. 3, as all subjects received the maximum number of 60 training sessions.

**Table 1:** Number of individuals that completed each training stage (either by reaching the completion criterion or by completing 60 sessions) and mean number of sessions required to complete each training stage.

Training stage	Age group	N	Number of sessions to complete training stage	
			Mean	Standard error
TS1	Young	4	4.8	0.5
	Adult	4	6	0
	Aged	3	3.7	0.7
TS2	Young	4	8.8	2.5
	Adult	4	13.8	5.1
	Aged	3	16.3	3.5
TS3	Young	4	35.5	8
	Adult	4	58.5	1.5
	Aged	3	54.3	3
TS4	Young	2	60	0
	Adult	2	60	0
	Aged	1	60	0

The analysis revealed a significant effect of the factor Training Stage (LMM: *F*_3,18.78_ = 130.14, *P* < 0.001), as the number of training sessions required to pass a stage increased as more sequence items were added during consecutive stages, assuming a significant cubic function (LMM: *t*_17.72_ = 5.67, *P* < 0.001). Pairwise comparisons confirmed that, although training performance in TS1 and TS2 did not differ significantly (Bonferroni post-hoc *t*-test: *t*_19.1_ = 2.60, *P* = 0.11), the number of training days required to pass TS3 was significantly higher compared to the former two stages (Bonferroni post-hoc *t*-tests: TS3 vs. TS1: *t*_19.1_ = 15.37, *P* < 0.001; TS3 vs. TS2: *t*_18.4_ = 12.54, *P* < 0.001), and higher yet again in TS4 (Bonferroni post-hoc *t*-tests: TS4 vs. TS3: *t*_21.2_ = 2.92, *P* = 0.049; comparisons to TS1 and TS2: both *P* ≤ 0.001).

The factor Age Group had no relevant influence on the number of training sessions required to pass a training stage across all four stages, as no age group showed a consistently lower or higher number of sessions than the other groups (LMM: *F*_2,6.65_ = 0.87, *P* = 0.462). However, the emerging interaction effect of the two factors (LMM: *F*_6,18.76_ = 2.62, *P* = 0.051) pointed towards differences between age groups within single training stages, confirmed by the subsequent simple-effect analysis; although subjects within the three age groups were equally successful in TS1 (ANOVA: *F*_2,10.5_ = 0.03, *P* = 0.97) and TS2 (ANOVA: *F*_2,10.6_ = 0.18, *P* = 0.84), this was not the case for TS3 (ANOVA: *F*_2,10.6_ = 5.46, *P* = 0.024). For TS3, young subjects performed significantly better than their adult counterparts (Bonferroni post-hoc *t*-test: *t*_9.08_ = 2.27, *P* = 0.049; the high variance within the aged group makes comparisons to this age group inconclusive, both *P* ≥ 0.346). In contrast, all three age groups were equally unable in completing TS4 within the 60 training sessions provided (ANOVA: *F*_2,12.1_ = 0.03, *P* = 0.97).

To obtain a measure of progress for TS4, we evaluated the highest daily performance score (correctly completed trials out of the ten daily trials) obtained halfway through the training stage, after 30 sessions of TS4 had been completed. This allowed us to include the data from the two young subjects that had to cease training before completing all 60 sessions of TS4. The analysis revealed that there were no statistically significant differences between the three age groups in this early performance level (Kruskal–Wallis test: *χ*_2_^2^ = 1.56, *P* = 0.459).

In summary, increasing the number of sequence items with each training stage led to an increase in the number of sessions required to acquire the sequence. Age differences were only notable for TS3, as young subjects were able to successfully complete this stage within fewer sessions than adult or aged subjects.

### Testing

Of the 12 pigeons that started the experiment, six completed all nine sessions of the Choice Test and sufficient sessions of the Memory Test to accumulate at least ten Memory Test trials. Given the low number of subjects, we analysed individual performance (see Tables 2, 3 and 4) and grouped subjects based on the number of sequence items they had been trained on instead of age groups. To facilitate the description and analysis of the tests, in addition to the sequence items used during training (i.e., sequence items 1 through 4), the novel items presented during the tests were numbered consecutively (i.e., sequence items 5 and 6). However, there was no qualitative difference to the latter two items.

**Table 2:** Percentage of trials (out of three trials) during which a subject chose the item that had appeared earlier in the sequence.

Subject	Age	Choice between sequence items					
		1 vs 2 (%)	1 vs 3 (%)	1 vs 4 (%)	2 vs 3 (%)	2 vs 4 (%)	3 vs 4 (%)
Yellow 23	7	100	67	–	100	–	–
Green 1076	6	100	67	–	0	–	–
DV 312	17	100	100	–	33	–	–
**Average**		**100**	**78**	**–**	**44**	**–**	**–**
Yellow 2	1	100	100	100	33	100	67
White 8174	7	100	100	100	0	100	100
Orange 11	8	100*	100	100*	50*	100	0
DV 573	15	100	100	100	100	100	67
**Average**		**100**	**100**	**100**	**46**	**100**	**58**

Individuals are listed in order of the number of items they received during training; those that only completed up to TS3 were not presented with choices including sequence item 4.

*Based on two trials instead of three.

**Table 3:** Percentage of Choice Test trials during which a subject chose the known item when the choice was between a known (sequence items 1, 2, 3, or 4, as applicable) and an unknown item (sequence items 5, 6, or 4, as applicable), or between two unknown items.

Subject	Age	Known option was			
		Item 1 (%)	Item 2 (%)	Item 3 (%)	Item 4 (%)
Yellow 23	7	100	67	70^$^	–
Green 1076	6	89	89	100	–
DV 312	17	89	56	50^$^	–
**Average**		**93**	**71**	**74**	**–**
Yellow 2	1	100	100	67	100
White 8174	7	100	83	83	67
Orange 11	8	80*	75^^^	60*	25^^^
DV 573	15	100	50	67	67
**Average**		**95**	**77**	**69**	**65**

Individuals are listed in order of the number of items they were trained on; those that only completed up to TS3 were not presented with sequence item 4 as a known option.

Percentages are based on nine trials for subjects Yellow 23, Green 1076 and DV 312, and on six trials for Yellow 2, White 8174 and DV 753.

^$^Based on ten trials. *Based on five trials. ^^^Based on four trials.

**Table 4:** Percentage of Memory Test trials (out of ten trials) during which a subject chose sequence item 1 before any other items (“1 first”), chose sequence item 2 directly after item 1 (“1 → 2”), chose sequence item 3 directly after item 2 (“2 → 3”), chose sequence item 4 directly after item 3 (“3 → 4”; only for those pigeons that had experienced training stage 4), chose the final trained items as its last choice (“3/4 last”), and when a subject completed the entire sequence of either three or four trained items in its correct order (“1 → 2 → 3 (→ 4)”).

Subject	Age	1 first (%)	1 → 2 (%)	2 → 3 (%)	3 → 4 (%)	3/4 last (%)	1 → 2 → 3 (→ 4) (%)
Yellow 23	7	80	40	30	–	30	30
Green 1076	6	70	30	10	–	10	10
DV 312	17	80	30	20	–	20	20
**Average**		**77**	**33**	**20**	**–**	**20**	**20**
Yellow 2	1	100^^^	64^^^	45^^^	73^^^	45^^^	45^^^
White 8174	7	100	70	50	30	10	10
Orange 11	8	67*	33*	33*	33*	33*	33*
DV 573	15	82^^^	36^^^	9^^^	18^^^	18^^^	0^^^
**Average**		**87**	**51**	**34**	**39**	**27**	**22**

Individuals are listed in order of the number of items they were trained on; those that only completed up to TS3 were not expected to visit sequence item 4.

*Based on three trials. ^^^Based on 11 trials.

#### Choice test

The Choice Test recreated the pairwise forced-choice tests conducted in previous studies on sequence learning to determine whether subjects had encoded the trained order. Table 2 shows the percentage of trials during which a subject chose the item that had appeared earlier in the sequence for each combination of trained sequence items. For the subjects experiencing up to TS3, although performance was numerically worse for choices between items 2 and 3, there was no statistically significant difference in performance between choices (Friedman test: *χ*_2_^2^ = 2.6, *P* = 0.273). However, for the four subjects that had reached TS4, whether or not the earlier item was chosen significantly depended on the presented options (Friedman test: *χ*_5_^2^ = 13.5, *P* = 0.019; Kendall’s *W* = 0.06). The pigeons showed a preference to choose item 1 when it was presented against any other item, and to choose item 2 in choices between items 2 and 4, but were less likely to choose the earlier item for choices between items 3 and 4 and for choices between items 2 and 3, as indicated by pairwise post-hoc comparisons (Bonferroni-corrected Durbin-Conover tests: comparing choices 2 vs. 3 and 3 vs. 4 to all other choices: all *P* = 0.057; all other comparisons: *P* = 1.0). Taken together, sequence item 1 was consistently recognised as the first item to attend to, whereas choices between other sequence items, including the final item, were less accurate.

In addition to the above, we examined pigeons’ overall encoding of the identity of trained sequence items by examining their choices when presented with a trained sequence item and a novel item. Table 3 shows the percentage of trials during which a subject chose the trained item for such choices. For those subjects that only completed TS3, the percentage of choice for the known item did not significantly change depending on which trained sequence item was presented (Friedman test: *χ*_2_^2^ = 1.64, *P* = 0*.*44). For subjects experiencing TS4, the percentage of choice for the known item also did not significantly change with the presented items (Friedman test: *χ*_3_^2^ = 6.26, *P* = 0*.*099), although the pigeons tended to choose trained items that appeared earlier in the sequence more often than trained items that appeared later, as shown in Table 3.

#### Memory test

As seen in Table 4, the overall tendency to visit items in the correct sequence was low (final column “1 → 2 → 3 (→ 4)”), with the highest success rate of 45% shown by the youngest subject, Yellow 2. To analyse performance, the following chunks within the sequence were considered: choosing sequence item 1 first, choosing item 2 directly after item 1, item 3 directly after 2, item 4 directly after 3 (if applicable), and choosing the final trained item last. For the group experiencing up to TS3, the percentage of trials during which a subject completed a chunk of one or two items in their correct order significantly decreased the later the chunk appeared in the sequence (Friedman test: *χ*_3_^2^ = 9.0, *P* = 0.029; Kendall’s *W* = 0.88). Pairwise post-hoc comparisons further confirmed that the percentage of choosing sequence item 1 first was significantly higher than choosing later chunks (comparisons of “1 chosen first” to any other option: Bonferroni-corrected Durbin-Conover test: *P* ≤ 0.006), and lowest for choosing sequence item 3 after item 2 and for avoiding the last item until the ultimate choice (comparison of “2 chosen after 1” to “3 chosen after 2” and to “3 chosen last”: Bonferroni-corrected Durbin-Conover test both *P* < 0.006; comparison of “3 chosen after 2” to “3 chosen last”: *P* = 1.0). Similarly, performance was significantly affected by the position of the chunk within the sequence for the subjects experiencing TS4 (Friedman test: *χ*_4_^2^ = 12.2, *P* = 0.016; Kendall’s *W* = 0.45). The percentage of choosing sequence item 1 first was significantly higher than choosing later chunks (comparisons of “1 chosen first” to “2 chosen after 3”, “3 chosen after 4” and “4 chosen last”: Bonferroni-corrected Durbin-Conover tests: all *P* ≤ 0.020; comparisons of “1 chosen first” to “2 chosen after 1”: Bonferroni-corrected Durbin-Conover test: *P* = 0.16). This indicates that predominantly only the first item was visited correctly, and pigeons were less likely to complete a chunk that appeared later within the sequence.

## Discussion

Aging can have detrimental effects on memory and cognition^1,2^. Here, we investigated potential age effects on pigeons’ memory and the encoding of serial order during sequence learning. Memory capacity declined noticeably with age, although not all aged pigeons suffered impairments when compared to their younger conspecifics, indicating some interindividual variability that emerges with age. The cognitive processes underlying sequence learning, however, appeared to be independent of age, as all pigeons performed in a way that indicated a mental encoding of sequence order.

The training data provided insight into an individual’s ability to remember sequences of increasing length. Lists of one and two items were acquired with relative ease by the pigeons regardless of age. However, as early as the three-item sequence, pronounced differences in acquisition rates emerged between the three age groups. Young pigeons learned the list within significantly fewer sessions than adult and aged pigeons, and half of the adult and aged individuals were unable to reproduce the sequence sufficiently within the session limit. No subject reached the learning criterion for the four-item sequence. The criterion was initially considered to be relatively high in an effort to avoid ceiling effects, and it was expected that at least the younger pigeons would be capable of reaching this standard, as the one-year-old pigeons in Terrace’s experiment achieved a comparable level of performance not only for four-item lists but also for five-item lists^5,6^. Considering that the current study employed an open-field paradigm, whereas previous studies used computerised tasks, it is difficult to pinpoint the cause of this discrepancy, which could range from visual aspects and differences in the mental representation of 2-dimensional and 3-dimensional stimuli (cf.^11–13^) to motoric aspects when pigeons have to move only their heads compared to their entire body from one stimulus to the next.

To acquire a measure of progress with the four-item list, we compared the highest reached daily score (correctly completed trials out of the ten daily trials) after the pigeons had received 30 of the 60 TS4 sessions. Although the highest scores were achieved by the youngest age group, older subjects did not fall far behind, and all subjects that had reached this training stage achieved similar thresholds for success as reported in other related studies (i.e., 30% correct in a single session^4^). The lack of clear age differences in TS4 despite observable differences in TS3 suggests that age does not affect pigeons’ memory performance uniformly. As is the case for humans^14^, there appear to be “high-performing” aging individual pigeons that preserve a high level of memory capacity (comparable to the performance of the least successful young subjects) and “low performing” aging individuals that show noticeable declines in memory. During a highly complex memory task such as the simultaneous-chaining task presented here, the decline is already noticeable at a relatively early age.

We incorporated two tests, a Choice Test and a Memory Test, to assess pigeons’ encoding of sequential order and infer potential differences regarding the cognitive mechanisms underlying sequence learning across the age groups. There was no indication in the obtained data to suggest performance differed between young, adult and aged pigeons in the two administered tests.

The Choice Test allowed for a comparison of the observed behaviour to previous studies. When presented with a forced choice between two list items, pigeons chose the first list item over items presented later in the sequence, and chose earlier items over the final list item, but they showed no preference for earlier sequence items when the choice was between two items presented in the middle of the list. Terrace interpreted these findings as evidence that his pigeons had chained responses to neighbouring items within a sequence, learning that a response to item *n* was only correct when preceded by a response to item *n-1*, but with no further reasoning about sequential order among the items^5^. He further asserted that the special positions of the first and the last sequence items were encoded separately, resulting in correctly ordered responses when the choices included one of these items, but disrupted performance when the choice did not include the first or the last item. The results from our Choice Test only replicate Terrace’s findings in part—our pigeons also showed their weakest choice performance during trials that included internal items of the sequence (2 vs 3 for those subjects that had trained with four items). However, performance was just as impaired when the choice included the two final items (3 vs 4, or 2 vs 3 for those subjects that had trained with three items), an observation that is directly contrary to Terrace’s argument of the final item assuming a “special role”. Indeed, reduced performance occurred primarily when the choice was between sequentially neighbouring items, regardless of whether the item pair occurred in the middle or at the end of the sequence. Item pairs that did not consist of immediately neighbouring items within the sequence reliably led to good performance (2 vs 4), which was at the same level of accuracy as any choices including item 1. The special role of the first item also mentioned by Terrace persisted in the current study as well, however, this was most likely due to overlearning—our pigeons had completed between 65 and 140 sessions each comprised of 10 trials (650–1400 trials that required a response to the first item). By comparison, Terrace mentioned that the most rapid acquisition of a 4-item sequence in his group’s line of experiments was achieved within 81 to 120 sessions of 80 trials (6480–9600 total trials^9^). Taken together, these results suggest that, instead of associatively chaining responses to neighbouring items, during the current experiment the pigeons encoded the order of items to some degree, although it might have been more in terms of items that appear “earlier” or “later” within the sequence than in terms of individual ordinal positions. The salience of the last item, which was so prominent in Terrace’s studies, was not confirmed here. However, Terrace’s pigeons were trained on five-item lists, which might have posed an additional level of cognitive difficulty to his subjects, resulting in good memory only of the first and last item but with little representation of the order of internal items. Instead, as noted by Scarf and Colombo, Terrace’s subjects relied on the display of all five list items together to be able to reproduce the order^4^. With shorter lists of four or three items, as used in this experiment, the cognitive load may have been reduced sufficiently to allow an encoding of the relative position of items as appearing earlier or later within the list.

The Memory Test allowed a further assessment of whether the observed behaviour supported the hypothesis proposed by Terrace regarding chaining of responses without an inherent concept of order^5^, or the more cognitively complex process proposed by Scarf and Colombo^4^. The most obvious result of this test was that item pairs further down the list were increasingly less likely to be chosen in their correct order. It is unlikely that this failure to adhere to the trained sequence stemmed from a lack of memory of the later items themselves—although the subjects had received less training sessions with later sequence items overall, all items were equally preferred over novel items in the pairwise-choice test. Instead, we observed that subjects abandoned the sequence as soon as their visit to the first item resulted in an absence of reinforcement. This observation is difficult to combine with Terrace’s account of chained responses, by which responding to one item automatically cues the response to the next. As the pigeon had to complete two baseline trials correctly before entering a Memory Test trial, it is unlikely that the sudden cessation in response behaviour was due to a lack of motivation or an inability to remember the sequence that the pigeon had completed successfully in the immediately preceding trial. Although unexpected, the observed behaviour indicates that pigeons had formed a concept of the goal of completing the sequence (to maximise reward) and acted in a planned way to achieve it. The pigeons did not perceive each item as an individual stimulus-outcome event, but indeed as part of a connected sequence. When the expected reward was not encountered in sequence item 1 during the non-reinforced test trials, this disruption was likely extrapolated to the entire sequence and the subjects changed their behaviour from the formerly goal-directed completion of the sequence to an unstructured search.

The combination of results of both tests makes it evident that the arguments put forward by Terrace^5^ are not compatible with the behaviour shown by our pigeons. Instead of acquiring simple response-response associations, the pigeons showed evidence of forming a representation of the order of list items, although this representation might be less sophisticated than proposed by Scarf and Colombo^4^, and instead limited to a concept of items that appear earlier and later in the sequence. As Terrace pointed out as well, the first item takes a special role, likely due to excessive overtraining. Furthermore, although the absolute length of the sequence that could effectively be memorised was reduced for some older pigeons, the ability to form a mental representation of order was preserved in all pigeons. Regardless of age, pigeons were able to perform in a way that was consistent with the task goal of maximising reward.

Thus, the current study showed that age can have a noticeable effect on the cognition of pigeons. As previously shown for humans^15,16^, aging does not impact all cognitive abilities uniformly, nor are all individuals affected to the same degree. It is possible to identify cognitive capacities that are highly susceptible to age for many species, such as memory, as shown in this study. Our study further supports that aging is an individual process, manifesting itself differently in “healthy-aging” or “poorly-aging” individuals. Longitudinal studies, focussing on intraindividual changes over time, will be an ideal follow-up to the comparisons between cohorts that are currently feasible to explore this area further. Although aging research is currently still limited to a few species, it is evident that age plays a crucial role in many aspects of animal cognition and behaviour. Identifying suitable paradigms, like the one presented in this study, to investigate such age effects in a wide range of species is the first step to closing the gap.
